# The Efficacy of the Charlson Comorbidity Index and Its Age-Adjusted Version in Forecasting Mortality and Postoperative Outcomes Following Isolated Coronary Artery Bypass Grafting

**DOI:** 10.3390/jcm14020395

**Published:** 2025-01-10

**Authors:** Ozgur Baris, Gozde Oksuzler Kizilbay, Canbolat Mert Holat, Mustafa Egemen Uzturk, Mustafa Canikoglu, Aysegul Durmaz, Oguz Omay, Sadan Yavuz

**Affiliations:** 1 Department of Cardiovascular Surgery, School of Medicine, Kocaeli University, 41001 Kocaeli, Turkey; mertholat1@gmail.com (C.M.H.); mustafa.canikoglu@kocaeli.edu.tr (M.C.); aysegul.durmaz@kocaeli.edu.tr (A.D.); oguz.omay@kocaeli.edu.tr (O.O.);; 2 Department of Pulmonary Disease, Mardin Kızıltepe Government Hospital, 47400 Mardin, Turkey; gozdeoksuzler@gmail.com; 3 School of Medicine, Kocaeli University, 41001 Kocaeli, Turkey; egemenuzturk@gmail.com

**Keywords:** Charlson comorbidity index, CABG, in-hospital mortality, postoperative complications

## Abstract

**Background/Objectives**: The Charlson Comorbidity Index (CCI) is designed for evaluating comorbidities and mortality risks, with the age-adjusted CCI (ACCI) combining age and comorbidity assessments. Despite its long-standing use, research on CCI’s application in cardiac surgery patients is limited. This study assessed the effectiveness of CCI and ACCI in predicting in-hospital mortality and post-surgery outcomes for patients undergoing isolated coronary artery bypass grafting (CABG). **Methods**: CCI and ACCI scores were derived from medical records between 2016 and 2022. Patient demographics, surgical techniques, and postoperative complications were documented. **Results**: Totally 393 patients [297 (75.6%) males, 96 (24.4%) females] with an average age of 65 years were included. Median CCI and ACCI scores were 1 (1–2) and 4 (3–5), respectively. In-hospital mortality occurred in 5.9% (n = 23) of cases, with CCI being an independent predictor (OR 1.865, 95% CI 1.117–3.116; *p* = 0.017). Both CCI and ACCI scores negatively correlated with preoperative EF (%) and positively correlated with ICU and total hospital stay, cardiopulmonary bypass time, and cross-clamp time. ACCI score also positively correlated with extubation time. Patients categorized by CCI comorbidity severity (no comorbidity, mild, moderate, severe) showed a significant increase in postoperative complications with increasing severity, including postoperative VT (*p* = 0.000), acute renal failure (*p* = 0.009), pneumonia (*p* = 0.007), and in-hospital mortality (*p* = 0.001). **Conclusions**: Both CCI and ACCI are prognostic indicators for in-hospital mortality in isolated CABG surgery patients, effectively predicting postoperative complications, extended ICU stays, and prolonged hospital stays. Implementing these scoring systems could enhance patient care and improve surgical decision-making.

## 1. Introduction

Ischemic heart disease has emerged as a major contributor to global mortality and morbidity in recent years, with its incidence rising annually. The World Health Organization (WHO) reported that ischemic heart disease claimed approximately 8.9 million lives worldwide in 2019 [1]. Coronary artery bypass grafting (CABG) surgery is a commonly employed and efficacious treatment for ischemic heart disease, especially in advanced cases requiring surgical intervention. However, the prevalence of ischemic heart disease is higher among older individuals, with age-related increases in mortality and complication rates [2]. This highlights the importance of classifying elderly patients as a high-risk group for surgical procedures. Additionally, the presence of coexisting conditions such as diabetes mellitus, preoperative renal insufficiency, and chronic obstructive pulmonary disease (COPD) is linked to increased mortality in CABG patients [3]. Many patients present with multiple comorbidities. A thorough assessment of patient risk factors before surgery and scoring comorbidities based on their collective impact, rather than individual evaluation, not only helps predict surgical complications and mortality but also plays a crucial role in determining the appropriate surgical approach. These approaches may include minimally invasive procedures, endovascular and hybrid techniques, and on-pump or off-pump coronary surgery. High-risk patients may benefit from minimally invasive methods, while standard surgical approaches may be suitable for lower-risk individuals [4].

The Charlson Comorbidity Index (CCI), developed in 1987, is a validated tool for scoring comorbidities and predicting mortality risk [5,6]. Initially created using one-year mortality data from internal medicine patients, the index was later validated in a cohort of breast cancer patients [7]. Since then, CCI has been applied to various conditions, including acute coronary syndrome, heart failure, cerebrovascular disease (CVD), COVID-19, and various surgical patient groups [7,8,9,10,11,12]. However, research evaluating CCI’s effectiveness in cardiac surgery patients remains limited [13,14].

This study aimed to assess the comorbidities identified during the preoperative period in patients undergoing isolated CABG surgery using the CCI and age-adjusted CCI (ACCI) scores. Additionally, it aimed to investigate the predictive impact of CCI and ACCI on in-hospital mortality and postoperative complications. The study’s findings are expected to contribute to enhancing preoperative risk assessment processes, personalizing surgical approaches, and improving patient management.

## 2. Materials and Methods

### 2.1. Study Population

A total of 393 patients aged 18 years and older who underwent isolated coronary artery bypass graft (CABG) surgery at Kocaeli University Faculty of Medicine Hospital between 2016 and 2022 were included in the study. Subjects were selected based on the availability of their baseline demographic information and the feasibility of calculating their CCI scores through retrospective analysis of hospital records. The study excluded patients with urgent and emergency CABG cases, patients with incomplete records, and those who had surgeries beyond isolated CABG (such as valve/multiple valve operations and combined coronary artery + valve procedures) during the same timeframe. Comorbidity information was extracted from patient medical files using ICD-10 codes.

The study recorded patients’ demographic details including age, gender, tobacco use history, and comorbidities. Surgical procedure characteristics were also documented, comprising the operation type (OPCAB: Off-pump coronary artery bypass, CPB: On-pump cardiopulmonary bypass), quantity of grafted vessels, CPB duration, cross-clamp time, and extubation time. Additionally, the research examined clinical progression indicators such as intensive care unit (ICU) duration, overall hospital stay, in-hospital mortality, and post-surgery complications (atrial fibrillation (AF), ventricular tachycardia (VT), cerebrovascular disease (CVD), acute renal failure (ARF), and pneumonia).

### 2.2. Charlson Comorbidity Index

The Charlson Comorbidity Index is a validated tool for assessing comorbidities and predicting mortality. In the calculation of the CCI, 19 comorbid conditions (myocardial infarction (MI), congestive heart failure, peripheral vascular disease, cerebrovascular disease, dementia, mild-to-moderate or severe chronic lung disease, rheumatologic disease, peptic ulcer disease, liver disease, diabetes mellitus (DM) with or without chronic complications, hemiplegia, renal disease, metastatic or non-metastatic malignancy, leukemia, lymphoma, and AIDS) were scored based on their severity, with weights ranging from 1 to 6. The total comorbidity score was calculated for each patient [5]. The ACCI score was calculated by adding 1 point for every decade over 40 years of age [6]. The CCI and ACCI scores are presented in Table 1. Total CCI and ACCI scores were categorized into four levels of comorbidity severity based on the patient’s comorbid conditions: 0 points (no comorbidities), 1–2 points (mild), 3–4 points (moderate), and ≥5 points (severe) [15].

### 2.3. Ethical Statement

The Kocaeli University Non-Interventional Clinical Research Ethics Committee granted approval for the research (Protocol code: KU GOKAEK-2024/18.06, Date of approval: 6 October 2024). Given the retrospective nature of the study, obtaining informed consent was not required. Nevertheless, measures were taken to protect patient privacy by anonymizing identities and reporting data in a manner that prevented individual identification.

### 2.4. Statistical Analysis

Statistical analyses were performed using IBM SPSS Statistics for Windows version 25.0 (SPSS, Chicago, IL, USA). The Kolmogorov–Smirnov test was used for the normality of numerical data. Categorical variables were expressed as numbers (percentage), while continuous variables were expressed as median (25th and 75th percentiles) or mean ± standard deviation (SD). Categorical variables were compared using the chi-square test. For variables with a normal distribution, *t*-tests were used, whereas the Mann–Whitney U test was used for non-normally distributed data in the univariate analysis of in-hospital mortality. Binary logistic regression analyses were performed to estimate predictors of in-hospital mortality in patients with isolated CABG. Correlations between CCI scores and postoperative clinical outcomes were assessed using Spearman’s correlation test. A Kruskal–Wallis or one-way ANOVA test was used for the comparison of groups according to comorbidity severity (CCI or age-adjusted CCI score classification) with respect to normal distribution. Statistical significance was defined as a two-sided *p*-value < 0.05.

## 3. Results

### 3.1. Demographic Characteristics

The study included 393 patients, of whom 297 (75.6%) were male and 96 (24.4%) were female, with a mean age of 65 ± 8 years. Among the patients, 191 (48.6%) were non-smokers, 202 (51.4%) had a history of smoking, and 95 (24.2%) were active smokers. At least one comorbidity was present in 370 (94.1%) patients. The most common comorbidity was diabetes mellitus (45.5%), followed by a history of preoperative MI (35.6%) (Table 2). 

The median CCI score was 1 (1–2), while the median ACCI score was 4 (3–5). According to the EuroSCORE classification, 376 patients (95.7%) were categorized as low-risk. Regarding surgical methods, 282 patients (71.8%) underwent on-pump (CPB) procedures, while 111 patients (28.2%) were treated using the off-pump (OPCAB) technique. Notably, 139 patients (35.4%) received three grafts, and 141 patients (35.9%) underwent four graft procedures. The median length of stay (LOS) in the intensive care unit (ICU) and total hospital stay were 4 days (3–5) and 8 days (7–11), respectively. The most common complications following isolated CABG were postoperative AF (n = 50, 12.7%), pneumonia (n = 40, 10.2%), and acute renal failure (n = 26, 6.6%). The in-hospital mortality rate of the study population was 5.9% (n = 23). Demographic, preoperative, and postoperative characteristics of the study population are shown in Table 3.

### 3.2. In-Hospital Mortality

In the univariate analysis of in-hospital mortality, it was found that the mortality group had significantly higher rates of comorbidities, such as MI (*p* = 0.000), CVD (*p* = 0.000), and chronic renal disease (CRD) (*p* = 0.000). The median CCI (*p* = 0.014) and ACCI (*p* = 0.009) were higher, the preoperative EF (%) (*p* = 0.000) was lower, and a higher proportion of patients had moderate and high EuroSCORE (*p* = 0.001) than the survivors. CPB times were significantly longer in the mortality group than in the group without in-hospital mortality (163.5 ± 80.5 vs. 120 ± 31.1, *p* = 0.02). Extubation time (15 vs. 9, *p* = 0.024) and length of stay in the ICU (6 vs. 4, *p* = 0.017) were also significantly longer in the mortality group. Additionally, in the mortality cases, the incidences of postoperative AF (*p* = 0.047), VT (*p* = 0.000), and ARF (*p* = 0.000) were significantly higher (Table 4).

Binary logistic regression analysis of in-hospital mortality revealed that the CCI score, preoperative EF (%), presence of postoperative ARF, and length of stay in the ICU were independent variables affecting mortality. The incidence of in-hospital mortality increased 1.865-fold (95% CI 1.117–3.116, *p* = 0.017) with a CCI score; 0.936 fold (95% CI 0.885–0.99, *p* = 0.02) with lower preoperative EF (%); 13.58-fold (95% CI 2.015–91.56, *p* = 0.007) in the presence of postoperative ARF; and 1.087-fold (95% CI 1.035–1.141, *p* = 0.001) with longer length of stay in the ICU (Table 5).

### 3.3. Classification of Comorbidity Severity

**Figure 1:**
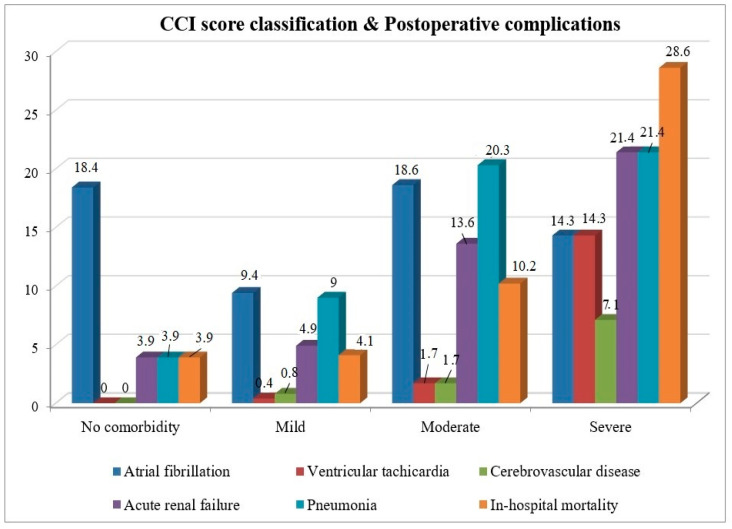
Postoperative complications and in-hospital mortality rates according to the classification of CCI scores (*p* values are respectively: atrial fibrillation *p*= 0.089; ventricular tachycardia *p* = 0.000; cerebrovascular disease *p* = 0.095; acute renal failure *p* = 0.009; pneumonia *p* = 0.007; in-hospital mortality *p* = 0.001).

This study demonstrated that the CCI score is an independent variable for in-hospital mortality in patients with isolated CABG. When the patients were divided into four groups according to CCI comorbidity severity (no comorbidity, mild, moderate, and severe), significant differences were observed between the groups in terms of the EuroSCORE risk category (*p* = 0.000), preoperative EF (%) values (*p* = 0.000), CBP durations (*p* = 0.001), postoperative ICU (*p* = 0.002), and total hospital length of stay (*p* = 0.000) (Table 6). It was found that preoperative EF (%) values decreased as comorbidity severity increased. Particularly in the group with severe comorbidities, a higher proportion of patients were classified into the moderate and high-risk categories of EuroSCORE compared to the other groups. As comorbidity severity increased, significant increases in postoperative VT (*p* = 0.000), ARF (*p* = 0.009), pneumonia (*p* = 0.007), and in-hospital mortality (*p* = 0.001) rates were observed (Figure 1). There was an increasing trend in the rates of postoperative pneumonia and in-hospital mortality associated with increased comorbidity severity. However, the difference was not statistically significant.

**Figure 2:**
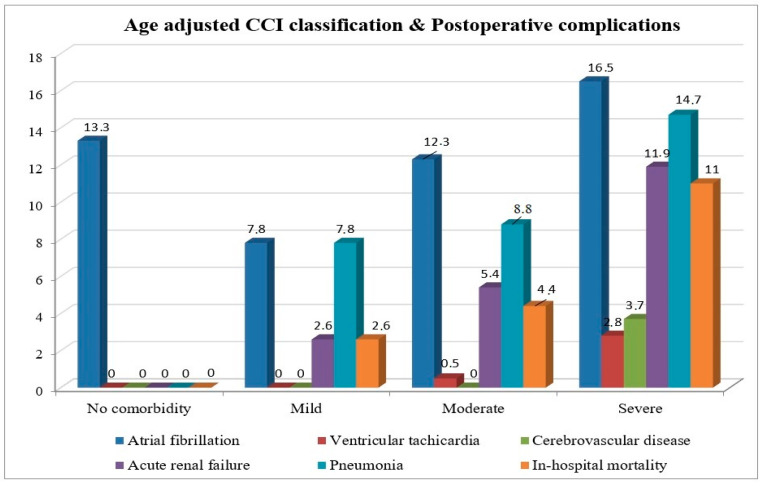
Postoperative complications and in-hospital mortality rates according to classification of age-adjusted CCI scores (*p* values are respectively: atrial fibrillation *p* = 0.23; ventricular tachycardia *p* = 0.2; cerebrovascular disease *p* = 0.015; acute renal failure *p* = 0.053; pneumonia *p* = 0.3; in-hospital mortality *p* = 0.053).

The comparison of the comorbidity severity groups defined by the ACCI score revealed that the incidence of postoperative cerebrovascular disease was significantly higher in the severe group (*p* = 0.015) (Figure 2). Furthermore, EuroSCORE, extubation time, and the length of stay in the hospital were significantly different between the groups (Table 7).

A negative correlation was observed between both CCI and ACCI scores and preoperative EF (%), whereas a positive correlation was found between postoperative ICU stay, total hospital length of stay, CPB time, and cross-clamp time. Additionally, a positive relationship was observed between the ACCI score and extubation time (Table 8).

## 4. Discussion

This study examined the effects of CCI and ACCI scores on in-hospital mortality and postoperative complications in patients undergoing isolated CABG surgery. CCI score was identified as an independent predictor of in-hospital mortality. In the subgroup analyses of the severity of comorbidities, significant differences were found among the groups in terms of EuroSCORE risk categories, preoperative EF (%) values, CPB duration, postoperative ICU stay, and length of hospital stay. In the classification of ACCI scores, significant differences were noted in EuroSCORE categories, extubation times, and hospital LOS. A significant relationship was found between the severity of comorbidities and postoperative complications in both CCI and ACCI classifications. Additionally, both scores correlated with postoperative ICU stay and/or length of hospital stay.

Despite recent technological improvements in preoperative patient care and risk management, CABG surgery is associated with mortality and morbidity. Advanced age and comorbidities are significant factors associated with postoperative mortality and morbidity after CABG [3]. Most patients present with multiple co-morbidities. Therefore, rather than evaluating the impact of each comorbidity individually, assessing them using a single scoring system is crucial for risk stratification, implementing preoperative measures, and selecting the appropriate surgical technique or alternative cardiovascular therapies for patients undergoing cardiac surgery.

The Charlson Comorbidity Index is a validated and easy-to-use tool that predicts mortality by assigning weighted scores to comorbidities [5]. The ACCI enables the combined evaluation of both age and comorbidities as risk factors. Although the CCI has been used for many years, studies evaluating its application in patients undergoing cardiac surgery are limited [13,14]. In a study by Minol et al., who examined the survival of patients undergoing mitral valve surgery, a high ACCI score was found to be significantly associated with 30-day and 1-year mortality rates [13]. Similarly, in another study that included patients undergoing mitral valve and aortic surgery, when patients were grouped according to severe and non-severe ACCI scores, no difference in in-hospital mortality rates was found between the groups. However, the 5-year and 10-year survival rates were higher in patients with lower scores [16]. In the literature, the only study evaluating the impact of ACCI on mortality in 5767 isolated CABG patients identified a significant relationship between ACCI, 5-year mortality, and hospital readmissions [14]. In this study, unlike Coyan et al., the aim was to evaluate the relationship between both CCI and ACCI and in-hospital mortality and morbidity, including postoperative complications, LOS-ICU, and LOS-hospital, in isolated CABG patients. Considering these aspects, our study provides a novel perspective on the existing literature.

The median CCI and ACCI scores in this study were 1 (1–2) and 4 (3–5), respectively, similar to the study by Coyan et al., with a mean ACCI score of 3.40 ± 1.75. Contrary to what is known in the literature, the in-hospital mortality rate of isolated CABG patients was found to be similar across sex and age groups. This finding may be partially related to the small sample size of the study population. In addition, preoperative comorbidities, such as MI, CVD, and CRD, were more frequent in the mortality group than in the surviving patients, which is consistent with the study by Coyan et al., involving isolated CABG patients [14]. In our study, the in-hospital mortality rate after CABG was 5.9%, and the median CCI and ACCI values were significantly higher in the in-hospital mortality group than in the survivors group. Multivariate analysis revealed that the CCI was an independent prognostic tool for in-hospital mortality in patients with isolated CABG.

CABG is associated not only with mortality but also with postoperative complications, prolonged ICU, and total hospital stay. In the literature, atrial fibrillation is reported as the most common postoperative complication after CABG, occurring in nearly 40% of patients, whereas it was observed in 12.7% of the patients in our study group [17]. The incidence of cerebrovascular disease, which can lead to postoperative mortality and prolonged hospital stay, has been reported to be 1.7% in one study, and, similarly, it was observed in 1% of our patients [18]. Acute renal failure is another postoperative complication that can occur after CABG, and, in our study, it was observed at a rate of 6.6%, which is higher compared to the 2–3% rate reported in the literature [19]. Since the frequency of “chronic renal disease” in the comorbidity distribution of the study population was 9.4% (n = 37), the postoperative acute renal failure rate was found to be higher than the literature at 6.6%, which can be explained by the demographic characteristics of the patient population.

The Charlson Comorbidity Index is typically used as a predictor of mortality. However, in our study, the impact of CCI on postoperative complications after isolated CABG was also evaluated, highlighting a strong and unique aspect of this study. A notable relationship was observed between higher CCI scores and the occurrence of postoperative complications, reflecting an increased comorbidity burden. Specifically, as comorbidity severity increased from mild to severe, there was a marked increase in the incidence of ventricular tachycardia (from 0.4% to 14.3%), acute renal failure (from 3.9% to 21.4%), pneumonia (from 3.9% to 21.4%), and cerebrovascular disease. Our study found that postoperative ICU and hospital LOS significantly increased with the severity of comorbidities. Similarly, Birim et al. reported that in patients undergoing surgery for non-small cell lung cancer, a moderate CCI score significantly predicted an increase in major postoperative complications and was associated with prolonged hospital stay [20]. In patients undergoing mitral valve surgery, higher ACCI scores were significantly associated with longer ICU stays and increased rates of new-onset hemodialysis due to acute kidney injury [13]. In a study investigating postoperative complications in CABG patients, a direct relationship between age and postoperative ICU stay, total hospital LOS, and number of postoperative complications was found, which is similar to the correlation analysis between ACCI and postoperative clinical outcomes in our study [21]. This finding parallels those of previous studies by Charlson et al., which highlighted the ability of ACCI to reflect the increasing comorbidity burden with age [8]. Although both CCI and ACCI have been shown to be prognostic indicators of in-hospital mortality, CCI has been found to be more predictive of postoperative complications. Therefore, CCI may provide a more effective assessment of the postoperative risk. On the other hand, since it is known that advanced age, as well as the presence of comorbidities, has been identified as a risk factor for mortality and postoperative complications after CABG, it can be thought that the use of ACCI, especially in older patients, may provide a more effective risk assessment.

Various tools have been used to assist clinicians in assessing mortality and morbidity risks after cardiac surgery. EuroSCORE, the Society of Thoracic Surgeons (STS), and the American College of Cardiology Foundation–Society of Thoracic Surgeons Collaboration on the Comparative Effectiveness of Revascularization Strategies (ASCERT) scores are commonly used scoring systems to predict mortality after cardiac surgery [16]. These methods involve dynamic cardiac function assessments and require expertise in their application [22]. In a study by Coyan et al., ACCI was compared with STS and ASCERT. It was reported that CCI could assist in determining mortality risk in situations where STS and ASCERT could not be applied [14]. In our study, the CCI and ACCI were compared with the EuroSCORE risk scores. Patients with moderate to high EuroSCORE risk were more frequent in the severe comorbidity group than in the other groups in both CCI and ACCI classifications. CCI and ACCI scores provide a more detailed comorbidity assessment as they include conditions such as malignancy, connective tissue diseases, and dementia, which are not evaluated using EuroSCORE II and STS scores. Additionally, they assess disease severity in conditions like cancer, diabetes, and liver disease. This enables the identification of risk factors that need to be monitored during the postoperative follow-up, leading to more accurate mortality predictions. Furthermore, the ability to objectively obtain data on existing comorbidities from national health data banks facilitates the practical application of these scoring systems.

### Limitations of Study

Our study had some limitations. Due to its retrospective design, single-center design, and relatively small study population, the findings may not be generalizable to the entire population. Since the study only included isolated CABG patients, a new prospective study is needed to evaluate the effectiveness of CCI and ACCI in non-isolated CABG cardiac surgeries or combined cases such as valve + CABG.

Despite these limitations, our study has several strengths. The number of studies evaluating the impact of CCI on mortality in cardiac surgery patients, particularly those undergoing isolated CABG, is limited. In our study, both CCI and ACCI were evaluated, providing risk stratification that included both age and comorbidities. Additionally, in-hospital mortality, postoperative complications, prolonged ICU stay, and hospital length of stay—important indicators of poor clinical outcomes—were also assessed.

## 5. Conclusions

In conclusion, both the CCI and ACCI have been demonstrated to be prognostic factors for in-hospital mortality in patients undergoing isolated CABG surgery. These scoring systems are also effective in predicting postoperative complications, prolonged ICU stays, and extended hospital stays, which are indicators of poor clinical outcomes. The use of these validated, easy-to-apply scoring systems, in addition to the EuroSCORE II and STS scores, could improve patient assessment processes, and patient management, and provide more precise guidance in surgical decision-making. By implementing detailed assessment phases, healthcare providers can make more informed decisions regarding patient care. For individuals with elevated CCI and ACCI scores, less invasive surgical techniques may be considered. Additionally, the Heart-team Joint Council may recommend hybrid approaches based on their collective decision-making process. Future studies should explore the integration of these systems into clinical practice in greater detail.

## Figures and Tables

**Table 1:** Calculation of Charlson Comorbidity Index scores and age-adjusted scores.

Comorbid Conditions	CCI Score	
Myocardial infarction	1	
Congestive Heart Failure	1	
Peripheral Vascular Disease	1	
Cerebrovascular Disease	1	
Dementia	1	
Chronic Pulmonary Disease	1	
Rheumatic Disease	1	
Peptic Ulcer Disease	1	
Mild Liver Disease	1	
Diabetes without chronic complication	1	
Diabetes with chronic complication	2	
Hemiplegia	2	
Renal disease	2	
Any malignancy without metastasis	2	
Leukemia	2	
Lymphoma	2	
Moderate or severe liver disease	3	
Metastatic solid tumor	6	
AIDS (exclude asymptomatic infection)	6	
Age	<40	+0
	41–50	+1
	51–60	+2
	61–70	+3
	≥71	+4
Max. comorbidity score	33	
Max. age-adjusted comorbidity score	37	

**Table 2:** Distribution of comorbidities in isolated CABG patients (%).

	n	%
Myocardial infarction	140	35.6
Congestive Heart Failure	31	7.9
Peripheral Vascular Disease	28	7.1
Cerebrovascular Disease	45	11.5
Dementia	2	0.5
COPD	30	7.6
Rheumatic disease	5	1.3
Peptic Ulcer	4	1
Chronic liver disease	9	2.3
Diabetes mellitus	179	45.5
Hemiplegia	4	1
Chronic renal disease	37	9.4
Solid organ tumor	23	5.9
Leukemia/Lymphoma	1	0.3
AIDS	0	0

**Table 3:** Demographic and operative characteristics of isolated CABG patients (n:393).

Demographic and Preoperative Characteristics		
Gender, n (%)	Women	96 (24.4%)
	Men	297 (75.6%)
Age	mean ± SD	65.95 ± 8.5
Smoking history, n (%)	Non-smoker	191 (48.6%)
	Current smoker	95 (24.2%)
	Former smoker	107 (27.2%)
Presence of any comorbidity, n (%)	(+)	370 (94.1%)
CCI	median (25th–75th percentile)	1 (1–2)
Age-adjusted CCI	median (25th–75th percentile)	4 (3–5)
CCI score classification	No comorbidity	76 (19.3%)
	Mild	244 (62.1%)
	Moderate	59 (15%)
	Severe	14 (3.6%)
Age-adjusted CCI classification	No comorbidity	3 (0.8%)
	Mild	77 (19.6%)
	Moderate	204 (51.9%)
	Severe	109 (27.7%)
EuroSCORE, n (%)	Low risk	376 (95.7%)
	Moderate risk	13 (3.3%)
	High risk	4 (1%)
Preoperative EF (%),	mean ± SD	56.1 ± 12.4
Surgical Procedures		
Surgical method, n (%)	OPCAB	111 (28.2%)
	CPB	282 (71.8%)
Number of grafts, n(%)	1	10 (2.5%)
	2	43 (10.9%)
	3	139 (35.4%)
	4	141 (35.9%)
	5	51 (13%)
	6	8 (2%)
	7	1 (0.3%)
CPB time, minute	mean ± SD	121.8 ± 35.5
Cross clamp time, minute	mean ± SD	70.4 ± 21.7
Extubation time, hour	median (25th–75th percentile)	9 (7–13)
Length of stay in ICU, day	median (25th–75th percentile)	4 (3–5)
Length of stay in hospital, day	median (25th–75th percentile)	8 (7–11)
Postoperative Complications		
	Postoperative AF	50 (12.7%)
	Postoperative VT	4 (1%)
	Postoperative CVD	4 (1%)
	Postoperative ARF	26 (6.6%)
	Postoperative pneumonia	40 (10.2%)
In-hospital mortality		23 (5.9%)

Abbreviations: SD: Standard deviation; CCI: Charlson Comorbidity Index; EF: Ejection fraction; OPCAB: Off-pump coronary artery bypass; CPB: On-pump cardiopulmonary bypass; ICU: Intensive care unit; AF: Atrial fibrillation; VT: Ventricular tachycardia; CVD: Cerebrovascular disease; ARF: Acute renal failure.

**Table 4:** Univariate analysis of in-hospital mortality in isolated CABG patients.

		In-Hospital Mortality (+)	In-Hospital Mortality (−)	*p*
Gender, n(%)	Women	9 (39.1%)	87 (23.5%)	0.09
	Men	14 (60.9%)	283 (76.5%)	
Age	mean ± SD	68.7 ± 9.1	65.8 ± 8.5	0.078
Smoking history, n (%)	Non-smoker	14 (60.9%)	177 (47.8%)	
	Current smoker	4 (17.4%)	91 (24.6%)	0.47
	Former smoker	5 (21.7%)	102 (27.6%)	
Comorbidity, n(%)		22 (95.7%)	348 (94.1%)	0.8
	Myocardial infarction	16 (69.6%)	124 (33.5%)	0.000
	CHF	3 (13%)	28 (7.6%)	0.34
	PVD	1 (4.3%)	27 (7.3%)	0.59
	CVD	8 (34.8%)	37 (10%)	0.000
	Dementia	0	2 (0.5%)	0.72
	COPD	2 (8.7%)	28 (7.6%)	0.84
	Rheumatic disease	0	5 (1.4%)	0.58
	Peptic Ulcer	0	4 (1.1%)	0.62
	Chronic liver disease	0	9 (2.4%)	0.45
	Diabetes mellitus	7 (30.4%)	172 (46.5%)	0.13
	Hemiplegia	1 (4.3%)	3 (0.8%)	0.1
	CRD	7 (30.4%)	30 (8.1%)	0.000
	Solid organ tumor	2 (8.7%)	21 (5.7%)	0.55
	Leukemia/Lymphoma	0	1 (0.3%)	0.8
	AIDS	NA	NA	NA
CCI	median (25th–75th percentile)	2 (1–3)	1 (1–2)	0.014
Age-adjusted CCI	median (25–75th percentile)	5 (3–7)	4 (3–5)	0.009
EuroSCORE, n (%)	Low risk	20 (87%)	356 (96.3%)	
	Moderate risk	1 (4.3%)	12 (3.2%)	0.001
	High risk	2 (8.7%)	2 (0.5%)	
Preoperative EF (%)	mean ± SD	43.9 ± 13.2	56.8 ± 11.9	0.000
Surgical method, n (%)	OPCAB	11 (47.8%)	100 (27%)	0.03
	CPB	12 (52.2%)	270 (73%)	
Number of grafts, n(%)	1	2 (8.7%)	8 (2.2%)	0.61
	2	3 (13%)	40 (10.8%)	
	3	7 (30.4%)	132 (35.7%)	
	4	8 (34.8%)	133 (35.9%)	
	5	3 (13%)	48 (13%)	
	6	0	8 (2.2%)	
	7	0	1 (0.3%)	
CPB time	mean ± SD	163.5 ± 80.5	120 ± 31.1	0.02
Cross clamp time	mean ± SD	85.8 ± 31.5	69.8 ± 21	0.073
Extubation time	median (25th–75th percentile)	15 (12–36)	9 (7–13)	0.024
LOS-ICU	median (25th–75th percentile)	6 (3–14)	4 (3–5)	0.017
LOS-hospital	median (25th–75th percentile)	7 (3–19)	8 (7–11)	0.14
Postoperative complications	Postoperative AF	6 (26.1%)	44 (11.9%)	0.047
	Postoperative VT	3 (13%)	1 (0.3%)	0.000
	Postoperative CVD	1 (4.3%)	3 (0.8%)	0.101
	Postoperative ARF	9 (39.1%)	17 (4.6%)	0.000
	Postoperative pneumonia	3 (13%)	37 (10%)	0.64

Abbreviations: SD: Standard deviation; CHF: Chronic heart failure; PVD: Peripheral arterial disease; CVD: Cerebrovascular disease; COPD: Chronic obstructive pulmonary disease; CRD: Chronic renal disease; CCI: Charlson Comorbidity Index; EF: Ejection fraction; OPCAB: Off-pump coronary artery bypass; CPB: On-pump cardiopulmonary bypass; LOS: Length of stay; ICU: Intensive care unit; AF: Atrial fibrillation; VT: Ventricular tachycardia; ARF: Acute renal failure.

**Table 5:** Binary logistic regression analysis of in-hospital mortality of isolated CABG patients.

Variable	OR [95% CI]	*p*
Age	1.021 [0.936–1.115]	0.63
EuroSCORE	1.8 [0.44–7.41]	0.9
CCI	1.865 [1.117–3.116]	0.017
Preoperative EF (%)	0.936 [0.885–0.99]	0.02
CPB time	1.018 [0.997–1.039]	0.087
Postoperative ARF	13.58 [2.015–91.56]	0.007
LOS-ICU	1.087 [1.035–1.141]	0.001

Abbreviations: EF: Ejection fraction; CPB: On-pump cardiopulmonary bypass; ARF: Acute renal failure; LOS: Length of stay; ICU: Intensive care unit.

**Table 6:** Comparison of demographic, preoperative, surgical, and postoperative characteristics of isolated CABG patients according to CCI score classification.

	No Comorbidity (n = 76)	Mild (n = 244)	Moderate (n = 59)	Severe (n = 14)	*p*
Women	17 (22.4%)	57 (23.4%)	19 (32.2%)	3 (21.4%)	0.51
Men, n (%)	59 (77.6%)	187 (76.6%)	40 (67.8%)	11 (78.6%)	
Age, years,	66.4 ± 8.9	65.8 ± 8.03	65.3 ± 9.8	68.9 ± 8.45	0.53
Smoking history, n (%)					0.36
Non-smoker	42 (55.3%)	116 (47.5%)	25 (42.4%)	8 (57.2%)	
Current smoker	14 (18.4%)	66 (327%9)	14 (23.7%)	1 (7.1%)	
Former smoker	20 (26.3%)	62 (25.4%)	20 (33.9%)	5 (35.7%)	
EuroSCORE, n (%)					0.000
Low risk	74 (97.4%)	240 (98.4%)	54 (91.5%)	8 (57.1%)	
Moderate risk	2 (2.6%)	3 (1.2%)	5 (8.5%)	3 (21.4%)	
High risk	0	1 (0.4%)	0	3 (21.4%)	
Preoperative EF%	60.2 ± 9.02	55.7 ± 12.2	54.7 ± 14.5	44.6 ± 12.5	0.000
Surgical method, n (%)					0.074
OPCAB	21 (27.6%)	61 (25%)	22 (37.3%)	7 (50%)	
CPB	55 (72.4%)	183 (75%)	37 (62.7%)	7 (50%)	
Number of grafts, n (%)					0.47
1	2 (2.6%)	6 (2.5%)	1 (1.7%)	1 (7.1%)	
2	4 (5.3%)	34 (13.9%)	4 (6.8%)	1 (7.1%)	
3	32 (42.1%)	75 (30.7%)	23 (39%)	9 (64.3%)	
4	25 (32.9%)	91 (37.3%)	22 (37.3%)	3 (21.4%)	
5	12 (15.8%)	32 (13.1%)	7 (11.9%)	0	
6	1 (1.3%)	5 (2%)	2 (3.4%)	0	
7	0	1 (0.4%)	0	0	
CPB time	115.9 ± 30.6	119.3 ± 30.6	136.2 ± 37.3	158.4 + 101.7	0.001
Cross clamp time	66.8 ± 23.4	70 ± 21.1	77.5 ± 22.6	73.6 ± 13.7	0.13
Extubation time	9 (7–14)	9 (7–13)	10 (8–14)	10 (6–14)	0.68
Length of stay in ICU	3 (3–4)	4 (3–5)	5 (3–7)	4 (3–5)	0.002
Length of stay in hospital	8 (7–9.8)	8 (7–10)	11 (8–14)	9 (4.6–13.3)	0.000

Abbreviations: EF: Ejection fraction; OPCAB: Off-pump coronary artery bypass; CPB: On-pump cardiopulmonary bypass; ICU: Intensive care unit.

**Table 7:** Comparison of demographic, preoperative, surgical, and postoperative characteristics of isolated CABG patients according to age-adjusted CCI score classification.

	No Comorbidity (n = 3)	Mild (n = 77)	Moderate (n = 204)	Severe (n = 109)	*p*
Women	1 (33.3%)	10 (13%)	55 (27%)	30 (27.5%)	0.076
Men, n (%)	2 (66.7%)	67 (87%)	149 (73%)	79 (72.5%)	
Age, years,	43.3 ± 4	58.3 ± 7.1	66.7 ± 7.2	70.5 ± 7.2	0.000
Smoking history, n (%)					0.085
Non-smoker	1 (33.3%)	32 (41.5%)	105 (51.5%)	53 (48.6%)	
Current smoker	1 (33.3%)	28 (36.4%)	47 (23%)	19 (17.4%)	
Former smoker	1 (33.3%)	17 (22.1%)	52 (25.5%)	37 (34%)	
EuroSCORE, n (%)					0.011
Low risk	3 (100%)	76 (98.7%)	200 (98%)	97 (89%)	
Moderate risk	0	1 (1.3%)	3 (1.5%)	9 (8.3%)	
High risk	0	0	1 (0.5%)	3 (2.7%)	
Preoperative EF %	58.3 ± 7.6	58.6 ± 12.2	56.3 ± 11.8	53.8 ± 13.3	0.067
Surgical method, n (%)					0.23
OPCAB	1 (33.3%)	19 (24.7%)	52 (25.5%)	39 (35.8%)	
CPB	2 (66.7%)	58 (75.3%)	152 (74.5%)	70 (64.2%)	
Number of grafts, n (%)					0.79
1	0	3 (3.9%)	3 (1.5%)	4 (3.7%)	
2	0	9 (11.7%)	25 (12.3%)	9 (8.3%)	
3	2 (66.7%)	24 (31.2%)	65 (31.9%)	48 (44%)	
4	1 (32.3%)	27 (35.1%)	75 (36.8%)	38 (34.9%)	
5	0	12 (15.6%)	30 (14.7%)	9 (8.3%)	
6	0	2 (2.6%)	5 (2.5%)	1 (0.9%)	
7	0	0	1 (0.5%)	0	
CPB time	99.5 ± 24.75	117.4 ± 115.5	120.4 ± 31.9	129.2 ± 44.02	0.17
Cross clamp time	52.5 ± 24.8	67.9 ± 63	70.7 ± 20.7	72.5 ± 20.8	0.43
Extubation time	12 (8–14)	8 (6–12)	9 (7–13)	10.5 (8–15)	0.023
Length of stay in ICU	5 (3–5)	3 (3–4)	4 (3–5)	4 (3–6)	0.314
Length of stay in hospital	8 (6–9)	8 (7–9)	8 (7–10)	10 (7–13)	0.002

Abbreviations: EF: Ejection fraction; OPCAB: Off-pump coronary artery bypass; CPB: On-pump cardiopulmonary bypass; ICU: Intensive care unit.

**Table 8:** Correlation analysis between CCI and age-adjusted CCI and pre and postoperative clinical outcomes.

Correlations Spearman’s Rho		Age	Preoperative EF %	LOS-ICU	LOS-Hospital	CPB Time	Cross Clamp Time	Extubation Time
CCI	Correlation Coefficient	0.015	−0.183 **	0.167 **	0.210 **	0.130 *	0.124 *	0.067
	Sig. (2-tailed)	0.76	0.000	0.001	0.000	0.029	0.037	0.194
	N	393	393	393	393	282	282	381
Age-Adjusted CCI	Correlation Coefficient	0.534 **	−0.173 **	0.123 *	0.189 **	0.152 *	0.129 *	0.129 *
	Sig. (2-tailed)	0.000	0.001	0.015	0.000	0.011	0.030	0.012
	N	393	393	393	393	282	282	381

Abbreviations: EF: Ejection fraction; LOS: Length of stay; ICU: Intensive care unit; CPB: Cardiopulmonary bypass; CCI: Charlson Comorbidity Index. * Correlation is significant at the 0.05 level (two-tailed); ** Correlation is significant at the 0.01 level (two-tailed).

## Data Availability

The data presented in this study are available upon request from the corresponding author. The data are not publicly available due to privacy regulations.

## Abbreviations

The following abbreviations are used in this manuscript:

ACCI	Age-adjusted Charlson Comorbidity Index
AF	Atrial fibrillation
AIDS	Acquired immune deficiency syndrome
ARF	Acute renal failure
CABG	Coronary artery bypass grafting
CCI	Charlson Comorbidity Index
CHF	Chronic heart failure
COPD	Chronic obstructive pulmonary disease
CPB	On-pump cardiopulmonary bypass
CRD	Chronic renal disease
CVD	Cerebrovascular disease
DM	Diabetes mellitus
EF	Ejection fraction
ICD	International Classification of Diseases 10th revision
ICU	Intensive care unit
LOS	Length of stay
MI	Myocardial infarction
OPCAB	Off-pump coronary artery bypass
PVD	Peripheral arterial disease
SD	Standard deviation
VT	Ventricular tachycardia
WHO	World Health Organization
